# Registration Difficulties

**Published:** 1908-12-26

**Authors:** 


### Nursing Administration.

### REGISTRATION DIFFICULTIES.

### VII.—CONCLUSION.

It is a grave consideration that legislation in respect of nurses will certainly be final, at least for this generation. It is not the custom in England as in America to permit laws to be dug up as it were by their roots from time to time and replanted. An Act for the registration of nurses, however imperfect, once passed will have to be faced, and whether for good or evil must be endured, practically in perpetuity. Whatever form legislation may take, it will set apart a class of nurses trained under certain conditions, and distinguished from all other nurses by the possession of a State certificate, and hence a certain proportion of the nurses eligible to obtain this distinction are eagerly agitating for it, believing it will prove of vital advantage to them in their career. A costly organisation will be necessary to carry into effect the proposed legislation. A Council of Registration must be elected and a Register of Nurses must be maintained. All women nursing the sick in this country will be divided into two sharply defined groups, those whose training is recognised and those whose training has not been recognised. As an inevitable consequence hospitals will also fall into two groups, those considered by the central body capable of training nurses and those debarred from this privilege. A brief *resumé* of the effects to be anticipated from this measure will serve to show the numerous interests concerned.

1. *The effect on the supply of nurses.* Registration has been proved in other countries to have exercised a marked influence in the direction of reducing the number of candidates for training; many women, otherwise well qualified for their nursing duties, are deterred from entering the profession by distrust of their ability to grapple with advanced theoretical studies and to pass examinations.

2. *The effect on institutions.* (a) Those "recognised" by the Registration Council. In many directions a general levelling up might be expected. But it is reasonably feared hospitals would be hampered by the unnecessary demands of an advanced theoretical curriculum. The present wholesome variety in training between one hospital and another would be reduced to a dead level, and while something would undoubtedly be gained, much that is priceless might be lost. (b) Institutions excluded from recognition. These would be not only in a very awkward position as regards attracting workers, but would be deprived of the smallest incentive to qualify their probationers. In other words the group of institutions which stands in real need of help and encouragement in the task of training women to undertake the duties of nurse would be altogether excluded by arbitrary regulations as to size from the scheme.

3. *The effect on nurses whose names were on the Register.*

(a) The bad nurses. Everyone with the smallest experience of registration work knows how impossible it is to keep undesirable people off a register, in view of their extreme eagerness to be provided with a passport of respectability. Their names once inscribed, the process of removing them is inevitably costly, tedious and uncertain. To prove even intemperance from a legal point of view is, as every superintendent knows, both difficult and dangerous. And it is very certain that whatever names are removed by the registrar on account of their owner's carelessness, it is not the names of undesirable nurses which will be effaced by this simple method.

(b) The indifferent nurses. The usual number of nurses who do well under average conditions, but are uncertain, tactless, or merely selfish, will be found on the register. Being registered nurses it is to be feared they will think pretty well of themselves, and that discredit will inevitably attach through their pretensions to their colleagues.

(c) The good nurses. Will good nurses fare any better than they do now under registration? It is more than doubtful. As now, they will be introduced to work through the medium of institutes, agencies, and medical men. There is not the smallest chance of any increase of fees, and the competition of unqualified women cannot be stopped by this or any other method it is possible to devise. The necessity for yearly verifying their names on the register will be a stumbling block to many and a charge upon all.

4. *The effect on nurses whose names are not on the Register.*

(a) The bad and indifferent will make a living as now, ready with a hundred plausible excuses for their exclusion, and favoured in certain quarters for that very reason.

(b) The good, often excluded from no fault of their own, will be undoubtedly liable to humiliation. The extent to which good nurses will hold back from the register, secure of lucrative employment without the State recognition, depends altogether on the extent to which the large hospitals oppose the register. There cannot be a doubt that doctors will continue to employ unregistered nurses as freely as they now employ those who are untrained.

5. *The effect on the public.* The public is absolutely indifferent in regard to the attainments of nurses. They cannot procure nurses save through recognised sources, and they could only be effectually safeguarded by the registration and inspection of nursing homes and institutes which by this measure are still left without regulation of any kind. Already people commonly show a tendency to dread the ministrations of nurses who "know too much," and some of those best qualified to judge believe that registration will deepen the popular notion that highly trained nurses are self-assertive and alarming, and that it will thus benefit the outsiders rather than the *élite*.

